# First two-stage robotic ALPPS in HCC patients with hepatic vein invasion: a step-by-step procedure from a clinical case

**DOI:** 10.1186/s12957-021-02170-0

**Published:** 2021-02-21

**Authors:** Ming-Gen Hu, Jin Wang, Zhu-Zeng Yin, Rong Liu

**Affiliations:** Faculty of Hepato-Pancreato-Biliary Surgery, Chinese PLA General Hospital; Institute of Hepatobiliary Surgery of Chinese PLA; Key Laboratory of Digital Hepatobiliary Surgery, PLA, Beijing, China

**Keywords:** Associating liver partition and portal vein ligation for staged hepatectomy, Hepatocellular carcinoma, Robotic, Portal vein anomaly, Future liver remnant

## Abstract

**Background:**

The associating liver partitioning and portal vein occlusion for staged hepatectomy (ALPPS) procedure is gaining interest because it brings hope to patients who cannot undergo radical surgical resection due to insufficient remnant liver volume. However, the indications and technical aspects of this procedure are still under debate. This report demonstrates the technical aspects of the first two-stage robotic ALPPS for HCC.

**Case presentation:**

A 55-year-old man with type II portal vein variation was diagnosed with hepatocellular carcinoma. Preoperative 3D reconstruction of the liver based on CT showed a future liver remnant/standard liver volume (FLR/SLV) of 24.45%. The ALPPS procedure was performed using the da Vinci Si system. At the first stage of the operation, we removed the gallbladder and ligated the right anterior branch of the portal vein and the right posterior branch. Following blocking of the hepatic hilum, the liver parenchyma was removed 1 cm away from the right side of the falciform ligament in an incision manner from the top to the bottom and from shallow to deep. The second-stage operation was performed on the 12th postoperative day with a FLR/SLV of 45.13%. During this step, the right hemiliver plus left medial section was separated and removed. Postoperative pathology showed a negative margin. The operative times were 195 and 217 min, respectively. Estimated blood loss was 250 and 500 ml, respectively. There was no need for transfusion or hospitalization in intensive care. The patient was discharged on the 6th postoperative day. Recovery was uneventful after both stages, and the patient did not present any sign of liver failure. Elevation of liver enzymes was minimal. The patient had no evidence of the disease 14 months after the procedure.

**Conclusions:**

The two-stage robotic ALPPS procedure is a safe and feasible technique for select patients with HCC.

**Supplementary Information:**

The online version contains supplementary material available at 10.1186/s12957-021-02170-0.

## Background

Traditional associating liver partitioning and portal vein occlusion for staged hepatectomy (ALPPS) is composed of two open operations, and the operation has always been controversial because of its high complication and mortality rates between 12 and 27% according to initial reports on ALPPS [1]. With the maturity and progress of laparoscopic hepatectomy, surgeons have proven that laparoscopic ALPPS is feasible [2]. Total laparoscopic ALPPS via the anterior approach was reported for primary hepatic carcinoma with cirrhosis in 2016 [3]. In this report, stage-one laparoscopic liver parenchyma dissection was considered to reduce intra-abdominal adhesion and surgical trauma, which was in line with the principle of damage control.

The first case of two-stage robotic ALPPS, reported by Spanish doctors in 2015, was a colon cancer liver metastasis [4], and two-stage robotic ALPPS has never been reported in patients with hepatocellular carcinoma (HCC).

## Case presentation

### Preoperative assessment

A 55-year-old male patient (body height 160 cm, body weight 58 kg, body surface area 1.6 m^2^ and standard liver volume 1132.3 ml) was admitted to our department on April 08, 2018, due to a focal liver lesion found on physical examination 1 month prior. Approval to perform ALPPS was obtained, as well as patient consent (Supplementary Materials).

Laboratory examination: alpha fetoprotein 3403.00 μg/l, Child-Pugh A level (total bilirubin 8.1 μmol/l, serum albumin 41.2 g/l, international standardized ratio 1.15, no ascites and hepatic encephalopathy) on April 7, 2018.

**Fig. 1:**
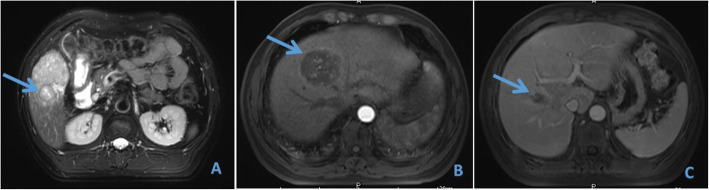
Preoperative MRI shows a tumour in the inferior segment of the right lobe of the liver (**a**) and another tumour at the border between the left and right lobes of the liver (**b**). In addition, the tumour thrombus in the right hepatic vein (**c**)

**Fig. 2:**
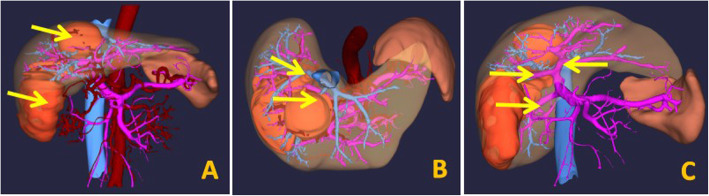
Preoperative liver 3D reconstruction shows tumours (**a**), tumours invading the middle and right hepatic veins (**b**) and the right anterior branch of the portal vein originating from the left branch of the portal vein (**c**)

Imaging examination: The patient underwent MRI examination in our hospital on April 4, 2018. The results showed two lesions in the liver. One of 59 × 53 mm was detected at the border between the left and right hemilivers, and another lesion of 51 × 109 mm was detected in the right posterior section. The portal venous-phase and delayed-phase scan showed a clear profile, filling defect in the right hepatic vein and ascending to the inferior vena cava; the middle hepatic vein was invaded by the tumour. In addition, the portal vein of this patient had type II variation (the main portal vein was first issued with the right posterior branch, which was further divided into the left branch and right anterior branch), which increased the difficulty of stage-one surgery (Figs. 1 and 2).

This patient was divided into III B (T3bN0 M0) at the TNM Classification of Malignant Tumours, which was not considered surgically treatable; Barcelona Clinic Liver Cancer (BCLC) stage is in stage B, and transcatheter arterial chemoembolization (TACE) treatment is recommended according to the guidelines. ALPPS has become a new choice for this patient to achieve R0 resection.

The patient was diagnosed with chronic hepatitis B for the first time after admission. The HBV DNA titre was 1.82 × 10^7^. Therefore, we administered entecavir 0.5 mg once a day as antiviral therapy, with no other special treatment before surgery.

### Surgical procedures

**Fig. 3:**
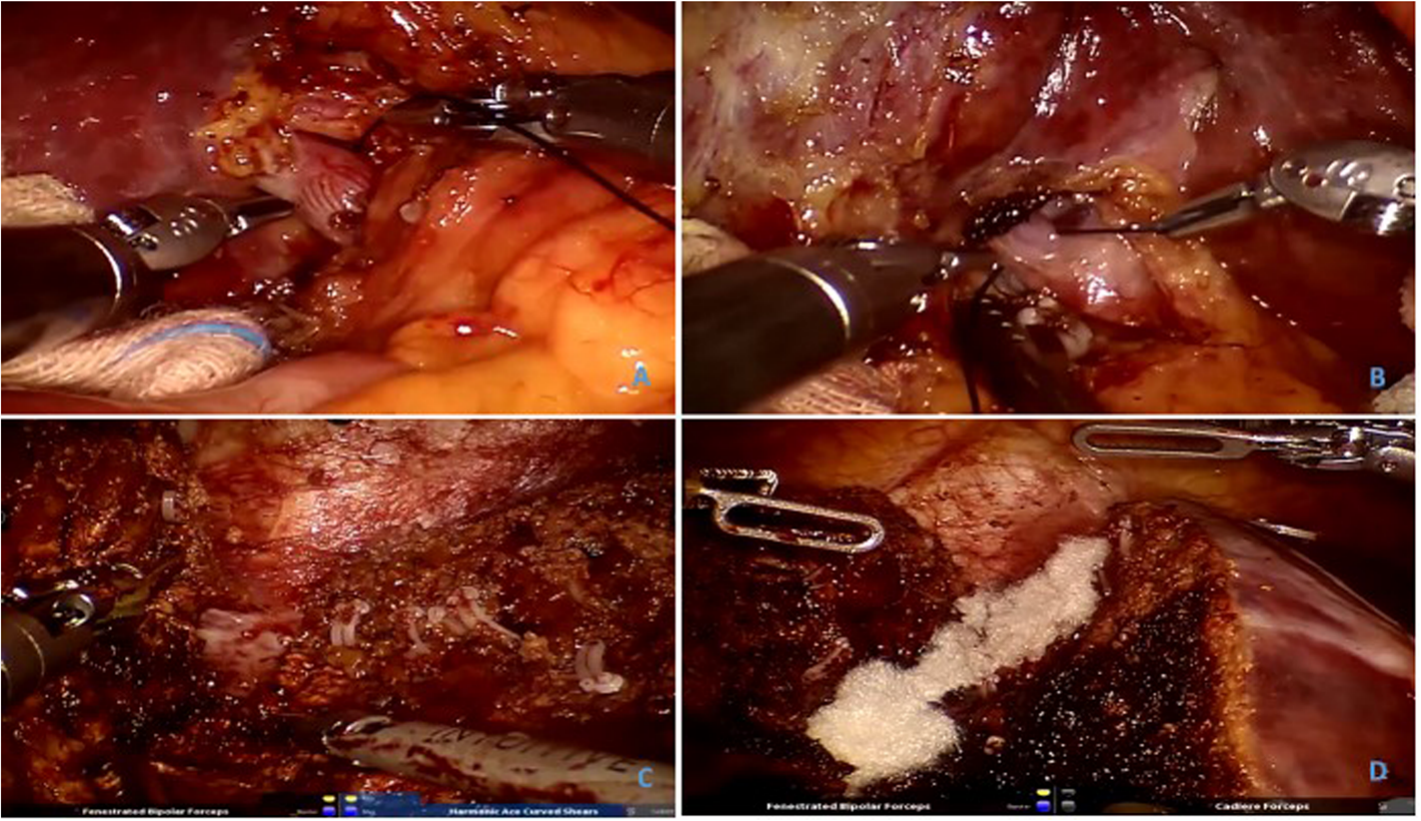
The first-stage operation findings. The right posterior portal vein (**a**) and the right anterior portal vein (**b**) were dissected and ligated. After liver parenchymal transection, the middle hepatic vein was separately isolated (**c**). A fibrillar absorbable haemosta was used for haemostasis (**d**)

The stage-one operation was performed on April 14, 2018. After successful anaesthesia, the patient was kept in a supine position, followed by routine disinfection, draping and insertion of a Veress needle and injection of carbon dioxide gas (14 mmHg). Then, the conventional operative hole was made for delivery laparoscopy and the operating instruments, which were operated on by the three-port method. The exploration showed no ascites, mild liver cirrhosis or giant hepatocellular carcinoma in the right lobe of the liver with partial growth outward. In addition, intraoperative ultrasonography revealed hepatocellular carcinoma located in S8 and S4 of the liver, as well as the left hepatic vein branch. With the marking of the splitting route of the liver within the above sites, the ligamenta teres hepatis and falciform ligament were cut off, in combination with the removal of the gallbladder. Subsequently, the right hepatic portal was dissected, and the right posterior and anterior branches of the portal vein were separated and ligated from the right incisura of the hepatic portal. The liver parenchyma was separated by an ultrasonic knife along the marked route. Following blocking of the hepatic hilum, the liver parenchyma was cut off 1 cm away from the right side of the falciform ligament in an incision manner from the top to the bottom and from shallow to deep. Afterwards, the S4 branch of the left hepatic vein and Glisson’s capsule were both ligated. With complete haemostasis, biological protein glue was sprayed on liver sections, followed by isolation with an anti-adhesion absorbable membrane. The drainage tube was placed and fixed, and the instrument and dressing were counted without mistakes. After that, the incision was sutured layer by layer. The hepatic portal was blocked twice during the operation, for 15 min each time (Fig. 3).

The gastric tube was removed on the first day after the operation, and the patient consumed fluids and walked on the second day after the operation. The liver volume was assessed on the sixth day after the first-stage operation. The volume of the liver in the left lateral lobe was 427.7 ml, an increase of 54.5%, and the FLV/SLV was 37.8%. Furthermore, the volume of the liver was re-evaluated on the tenth day after the operation. The volume of the left lateral lobe was 511 ml, which was an increase of 85%, and the FLV/SLV reached 45.13%.

**Fig. 4:**
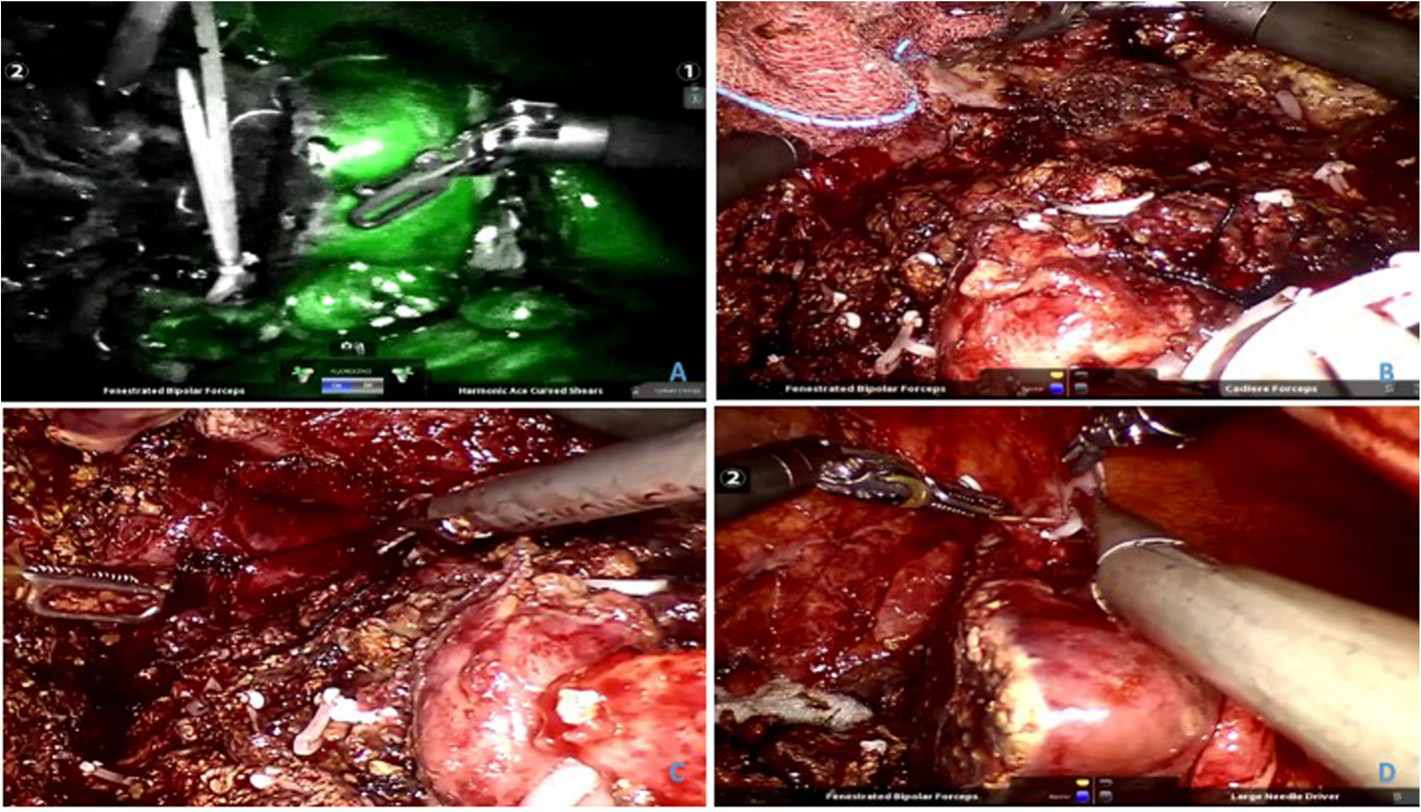
The second-stage operation findings. ICG was applied with a successful display of fluorescence in the left lateral section, the right liver and left medial section were not shown (**a**), the middle hepatic vein was transected (**b**) and the right hepatic vein (**c**), in the end, fixed the remaining liver (**d**)

The second-stage surgery was performed on the twelfth day after the first-stage surgery. With successful anaesthesia, the patient was kept in a head-high and feet-low lithotomy position. After routine disinfection, draping and insertion of a Veress needle and injection of carbon dioxide gas (pressure of 14 mmHg), the conventional operative hole was established for the placement of robot-assisted laparoscopic lenses and operating instruments, which were operated by the five-port method. The puncture device was arranged in a C shape. Exploration under the microscope showed a small amount of ascites, new adhesion of the omentum around the hepatic portal, slight tissue oedema, obvious enlargement of the left lateral lobe of the liver, no obvious change of the right lobe of the liver and its internal tumour and separation of the left external lobe splitting incision. Following separation of adhesions, the greater omentum adhering to the right lobe tumour was dissected, followed by dissection of the right hepatic ligament, separation of the right hepatic artery and cutting off after ligation. Intravenous injection of ICG (5 mg) was applied with successful development of the left lateral lobe; however, the right third lobules did not develop. Afterwards, the right anterior and posterior branches of the portal vein were separated, and the right hepatic duct was separated, clipped and then removed. Right and upward separation was carried out from the liver incision along the inferior vena cava surface. After ligating the short hepatic vein and separating the root of the middle hepatic vein, the left hepatic vein was clipped and cut following protection. Subsequently, the right hepatic vein was separated from the right side, and the right hepatic vein was cut apart, followed by ligation of the right inferior phrenic vein. Part of the right coronary ligaments that were not disconnected was then separated, and the right third lobules were removed. After a small incision was made in the lower abdomen, the specimen was extracted, the incision sutured and the pneumoperitoneum re-established. Finally, with complete haemostasis of the wound, the incision was irrigated, and two drainage tubes were placed. In the last step, the incision was closed layer by layer after confirmation of the instrument and dressings (Fig. 4).

Surgical results: The duration of the first and second operations lasted 191 and 227 min, respectively. Intraoperative bleeding was 50 ml, and there was no blood transfusion in either operation. Postoperative pathological results were reported as moderate and poorly differentiated hepatocellular carcinoma, without tumour tissues at the margin of incision.

### Changes in functional liver remnant volume and liver function

**Fig. 5:**
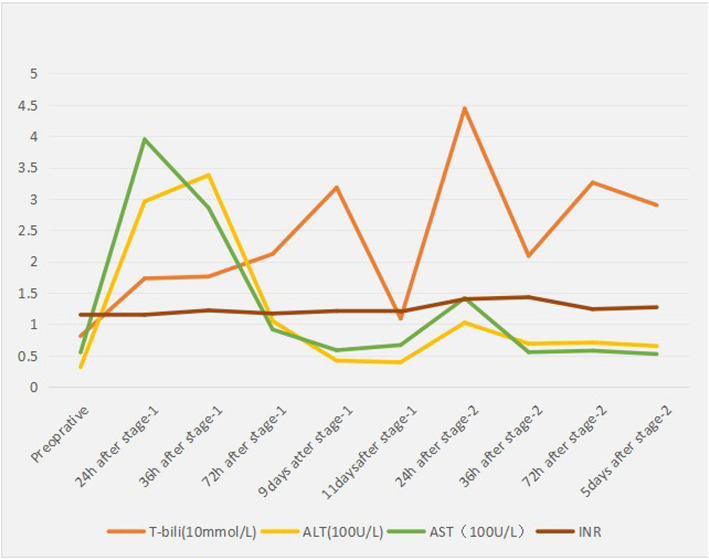
The total bilirubin (T-bili), ALT, AST and INR of patients at the perioperative stage

**Fig. 6:**
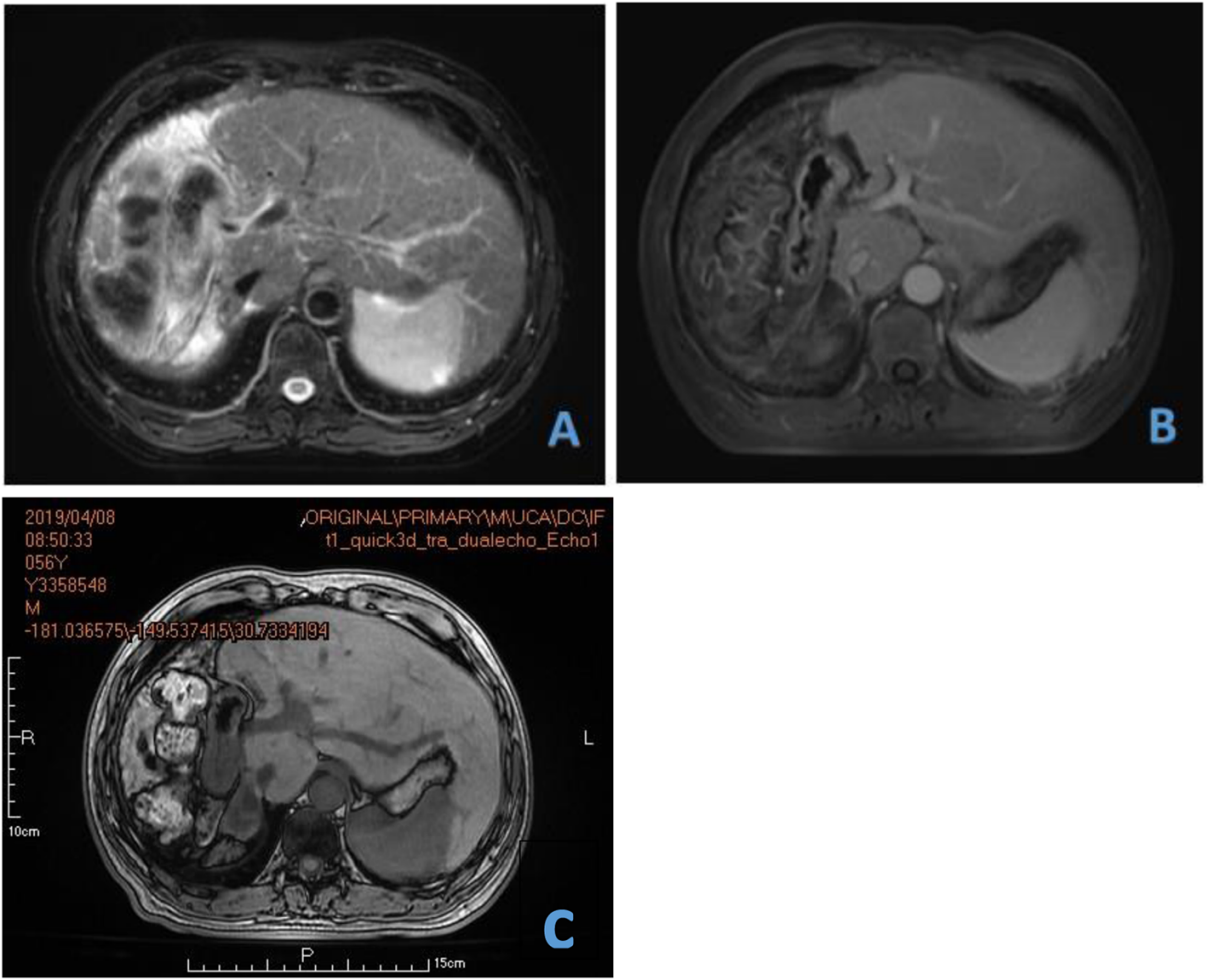
One month (**a**), 5 months (**b**) and 11 months (**c**) MRI after the operation

Before stage one, we performed 3D reconstruction of the CT results by using the Hisense Computer-Aided Surgery system, and the liver volume of the patients was 1267.4 ml. If the right and left hepatic lobes were removed along the margin of the tumour capsule, the remnant liver volume was 276.9 ml, and the RLV/SLV was 24.45%. After 11 days of stage-one surgery, we performed 3D reconstruction again. The patient’s left lateral section of the liver was 511 ml, which was an increase of 85%, and his future liver volume over the standard liver volume was 45.1% (Figs. 5 and 6). The last laboratory examination was on March 11, 2019. Elevation of liver enzymes was minimal, the HBV DNA titre was 36.7 IU/ml and alpha fetoprotein was 63.37 μg/l.

## Discussion

The development of ALPPS surgery has brought the hope of radical surgery to more than 1000 patients with giant liver tumours [5]. According to the 2014 report of the international ALPPS registration organization, 70% of cases are patients with colorectal liver metastases [1]. This is partly because liver resection in Western countries mainly refers to colorectal liver metastases and partly due to doubts about whether patients with hepatocellular carcinoma can benefit from ALPPS [6].

In 2016, the University of Hong Kong analysed 38 HCC patients who underwent ALPPS surgery. They all successfully underwent two operations, and the 90-day mortality rate was 7.1%, comparable to 8% for colorectal liver metastases. The average FLR/SLV ranged from 24.2 to 38.5% over a median of 6 days. This report demonstrates the efficacy and safety of ALPPS in treating HCC and emphasizes the importance of case selection [7].

The current clinical study suggests that independent factors for severe complications after ALPPS were red blood cell transfusion, stage-one operation taking more than 300 min and being over 67 years of age. Based on Hong Kong’s experience. We believe that ALPPS should be used only in patients with liver function Child-Pugh A levels to treat hepatocellular carcinoma [8–11].

Extensive experience with laparoscopic liver resection suggests that laparoscopy may reduce operative severity and complications such as blood loss, adhesion and bile leakage [12]. Machado and colleagues believed that laparoscopic ALPPS, as an easy solution for adhesions and difficulties that may be encountered during the second stage, is feasible and does not appear to be inferior to the open approach in experienced hands [13, 14].

The Da Vinci robotic surgery system can provide a better surgical field and flexible robot arms, reducing the difficulty of surgery and better avoiding damage to bile ducts and important blood vessels during the process of portal vein ligation and liver parenchymal detachment [15]. The perioperative outcomes of robotic hepatectomy are equivalent to those of laparoscopic hepatectomy and may even result in superior outcomes compared with laparoscopic hepatectomy [16].

In this patient with portal vein variation, the robot system assisted the operator in completing the operation successfully, and the patient recovered well after the operation. The Da Vinci robotic surgery system is a reliable choice for ALPPS. However, due to the limited number of cases, more cases need to be involved for observation in a relatively longer period of time.

## Conclusions

Two-stage robotic ALPPS procedure is a feasible technique for selected patients with HCC. It is imperative to design multicentre randomized controlled trials to assess the safety and efficacy of all robotic ALPPS in some selected HCC patients.

## Supplementary Information

**Additional file 1:** Supplementary Materials

## Data Availability

All data generated or analysed during this study are included in this published article.
