# Supplementary figures and images for: First two-stage robotic ALPPS in HCC patients with hepatic vein invasion: a step-by-step procedure from a clinical case
Source: World J Surg Oncol. 2021 Feb 21;19:58. doi: 10.1186/s12957-021-02170-0 (PMC7898755; doi:10.1186/s12957-021-02170-0)

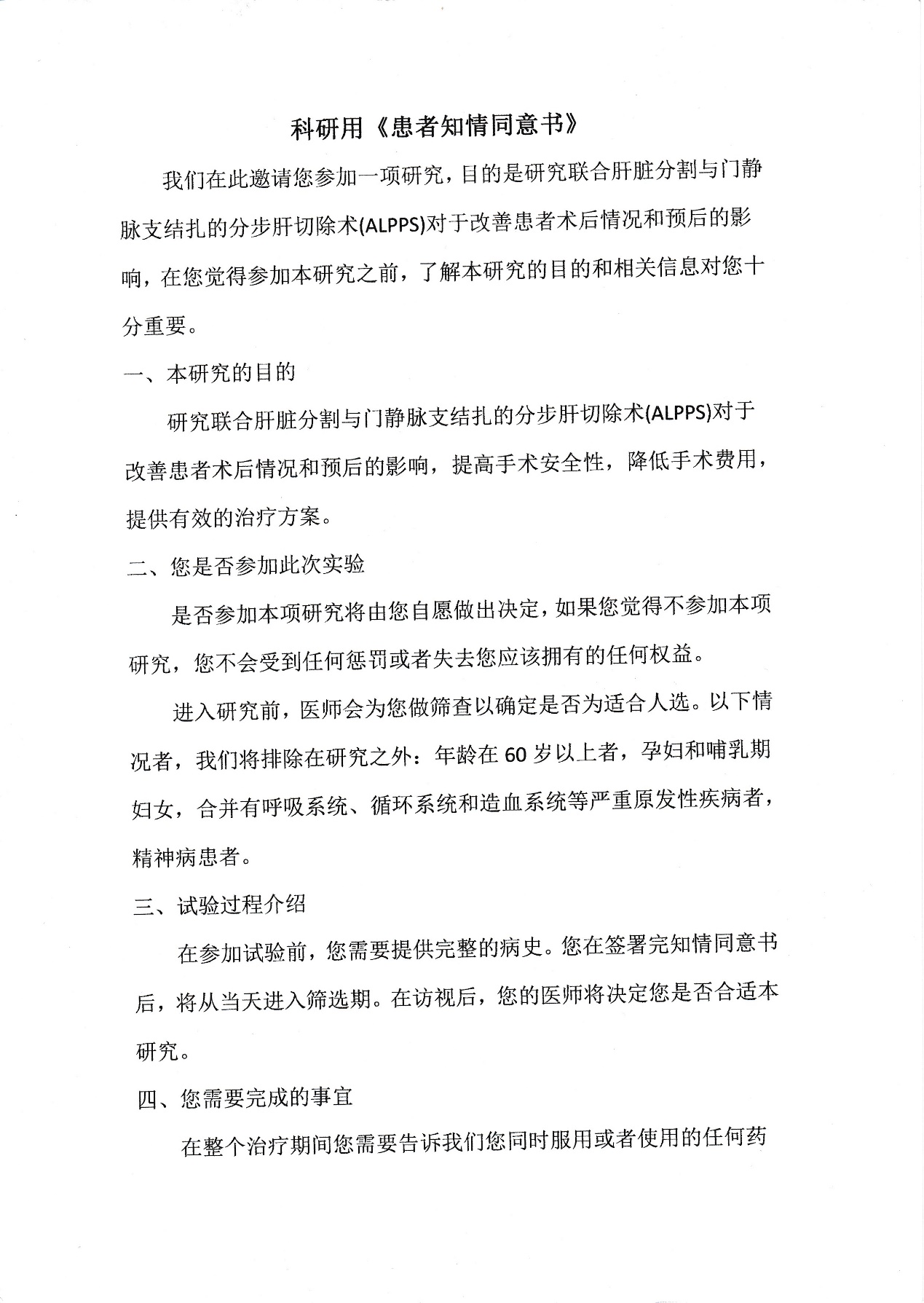


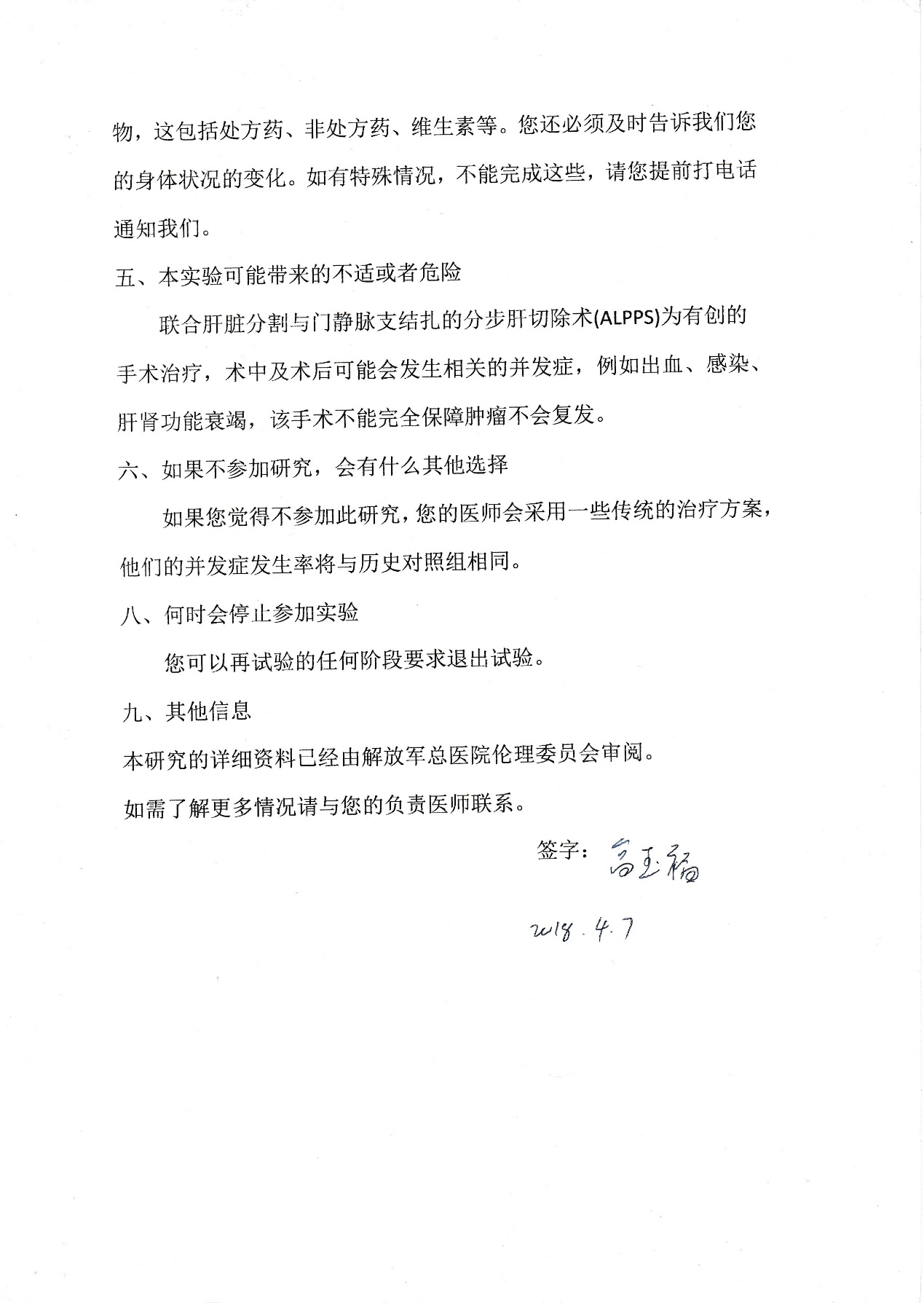


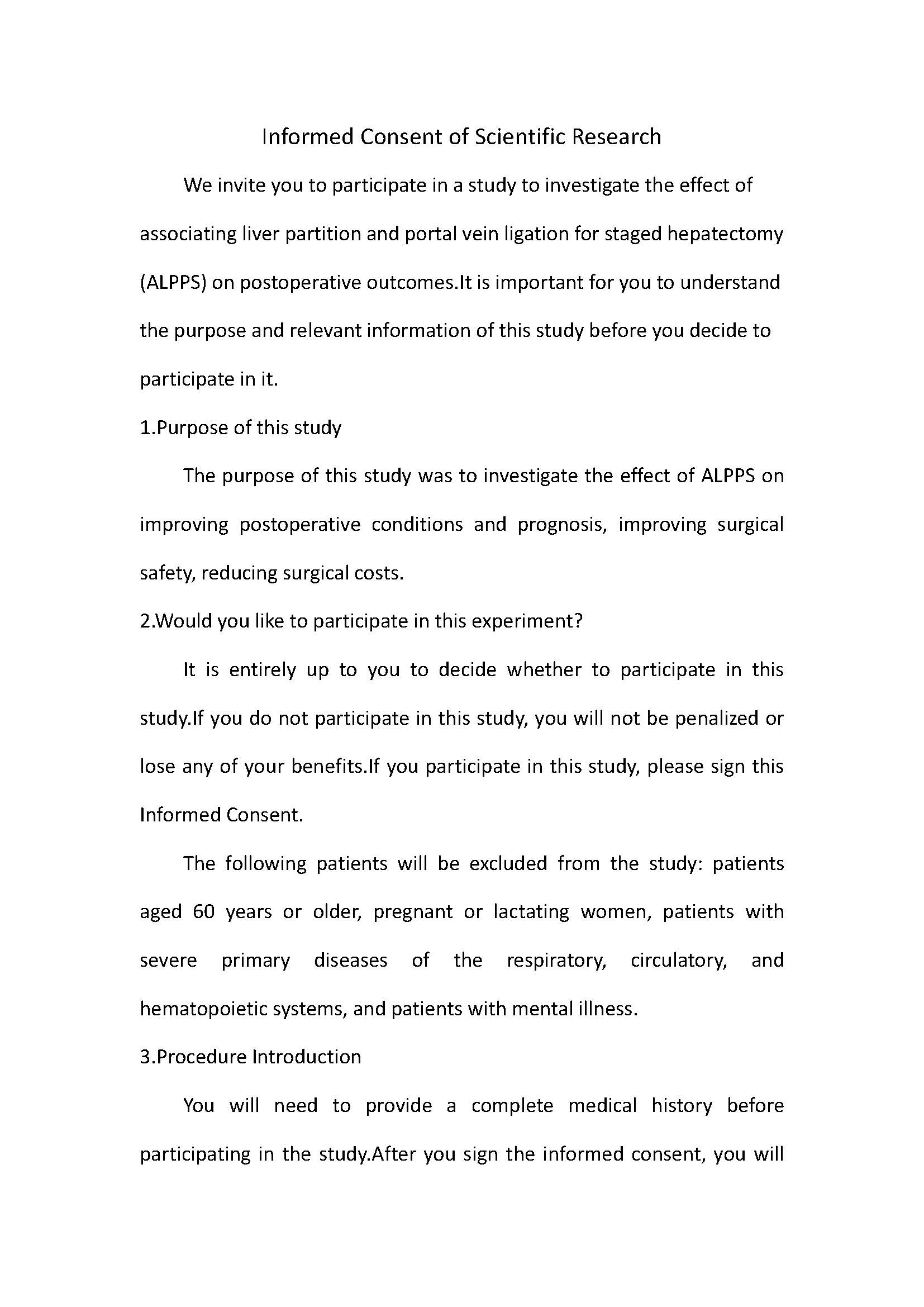


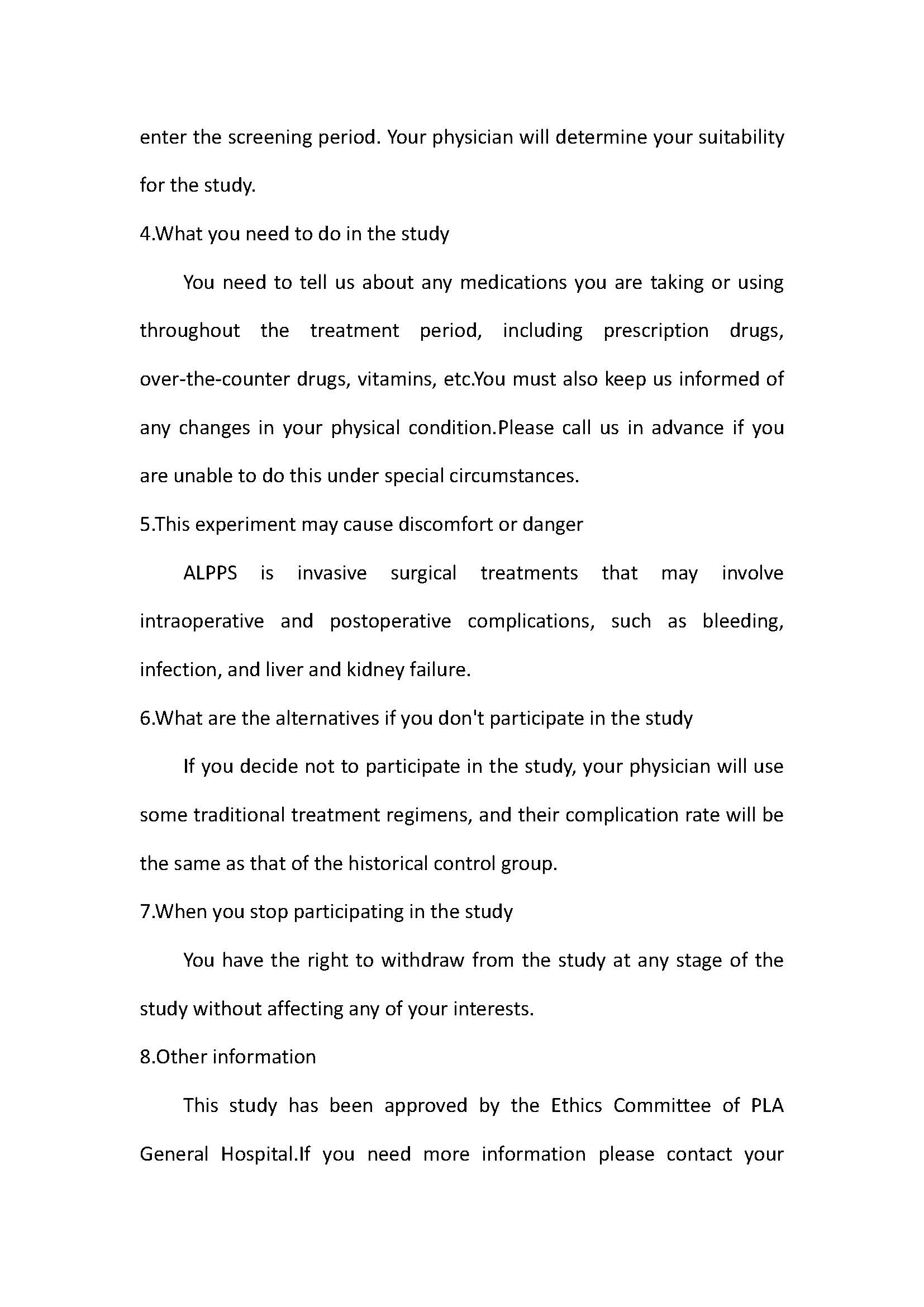


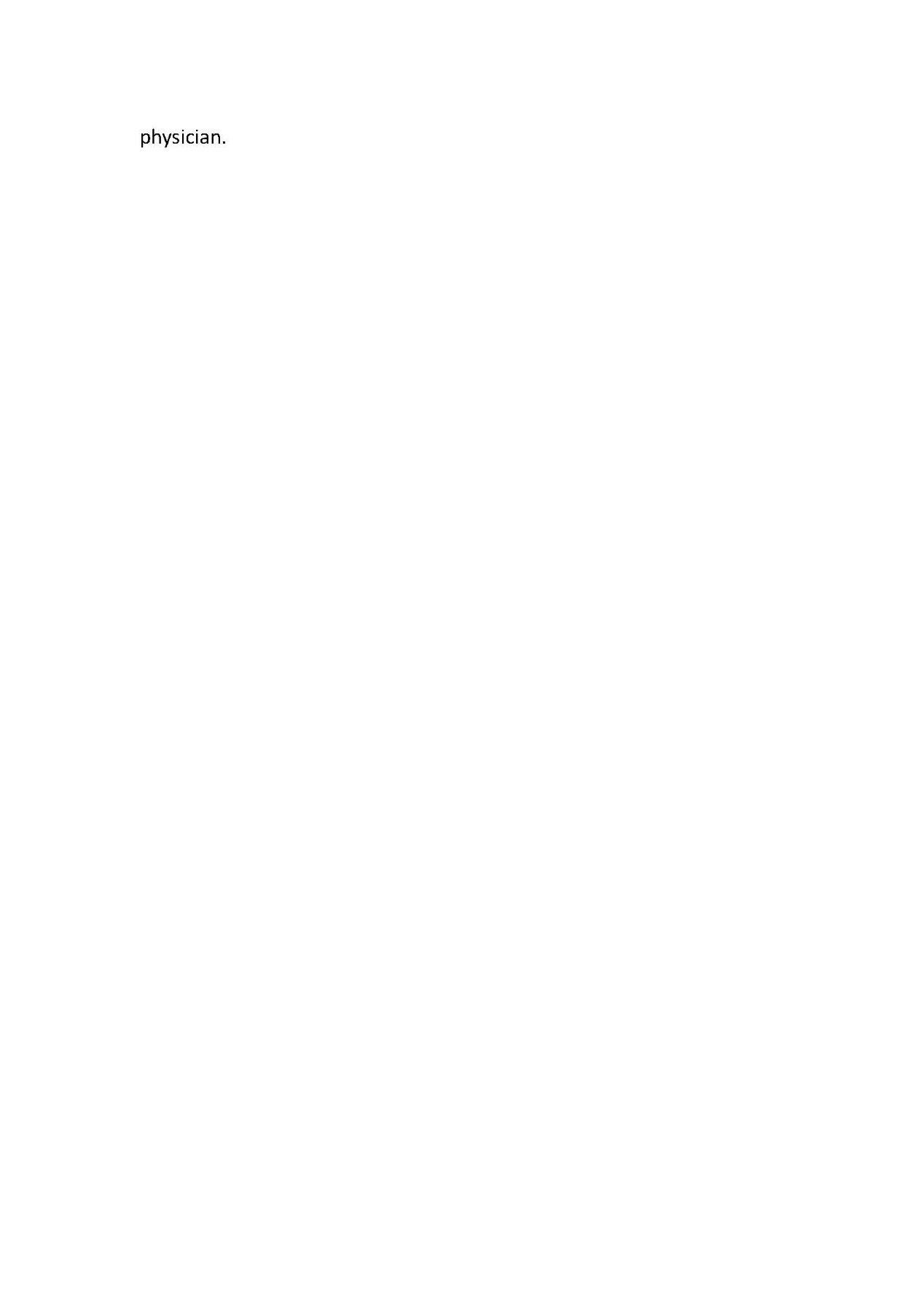

Supplement: Supplementary file 1 — Additional file 1: Supplementary Materials [file 12957_2021_2170_MOESM1_ESM.docx]
